# Regulatory B Cells in Pregnancy: Lessons from Autoimmunity, Graft Tolerance, and Cancer

**DOI:** 10.3389/fimmu.2017.00172

**Published:** 2017-02-17

**Authors:** Ruth Marian Guzman-Genuino, Kerrilyn R. Diener

**Affiliations:** ^1^Experimental Therapeutics Laboratory, School of Pharmacy and Medical Science, Hanson Institute and Sansom Institute for Health Research, University of South Australia, Adelaide, SA, Australia; ^2^Robinson Research Institute and Adelaide Medical School, University of Adelaide, Adelaide, SA, Australia

**Keywords:** regulatory B cells, regulatory T cells, autoimmunity, cancer, pregnancy, tolerance, estrogen, progesterone

## Abstract

The success of pregnancy is contingent on the maternal immune system recognizing and accommodating a growing semi-allogeneic fetus. Specialized subsets of lymphocytes capable of negative regulation are fundamental in this process, and include the regulatory T cells (Tregs) and potentially, regulatory B cells (Bregs). Most of our current understanding of the immune regulatory role of Bregs comes from studies in the fields of autoimmunity, transplantation tolerance, and cancer biology. Bregs control autoimmune diseases and can elicit graft tolerance by inhibiting the differentiation of effector T cells and dendritic cells (DCs), and activating Tregs. Furthermore, in cancer, Bregs are hijacked by neoplastic cells to promote tumorigenesis. Pregnancy therefore represents a condition that reconciles these fields—mechanisms must be in place to ensure maternal immunological tolerance throughout gravidity to allow the semi-allogeneic fetus to grow within. Thus, the mechanisms underlying Breg activities in autoimmune diseases, transplantation tolerance, and cancer may take place during pregnancy as well. In this review, we discuss the potential role of Bregs as guardians of pregnancy and propose an endocrine-modulated feedback loop highlighting the Breg–Treg–tolerogenic DC interface essential for the induction of maternal immune tolerance.

## Introduction

Pregnancy is a remarkable biological process wherein the mother carries an offspring that is semi-allogenic, that is, half of its genome is derived from the foreign paternal parent. It imposes significant physiological stress, requiring the mother’s body to undergo immense functional changes. Pregnancy in mammals represents a unique compromise in the context of immunity—the maternal immune system must accommodate the semi-allogenic fetus by dampening its immune responses to maintain a state of immunological tolerance throughout gravidity, while retaining the capacity to identify pathogens and destroy them with appropriate control of resultant inflammation. This dichotomy necessarily requires a fine and highly regulated balance between immune tolerance and immune activation.

In pregnancy, the immune profiles of dendritic cells (DCs), natural killer cells, macrophages, and T cells are by necessity modified (1–4). Recently, it has been shown that the B cell profile is also modified in response to the immunological needs of the mother during pregnancy (5). The role of B cells in pregnancy holds much interest, especially in light of the recent identification of a B cell subset capable of negative regulation, that is, can suppress immune inflammation. Due to their unique role amongst the B cell types, this subset is aptly designated as ‘regulatory B cells’ or ‘Bregs.’

Regulatory B cells are being extensively studied in three major fields: autoimmunity, transplantation tolerance, and cancer. Seemingly different, conditions in these fields are in fact outcomes of immune dysfunction. Whereas autoimmune diseases reflect an inadequate ability to control an overactive immune response to self, development of cancer results from the enthusiastic suppression of the immune system thus allowing a mutated self to survive and proliferate. The role of Bregs as negative regulators of the immune system varies in these different contexts. As mediators of immune suppression, they have been implicated in the cessation of autoimmune diseases and graft tolerance as the expansion of the Breg population is highly correlated with amelioration of the disease and maintenance of tolerance (6). On the other hand, Bregs have been tagged as pro-tumorigenic due to their capacity to inhibit the action of effector immune cells whose purpose is to identify and eliminate cancer cells, hence providing a favorable environment for the tumor to grow (7, 8).

As pregnancy requires selective immunological tolerance, this condition may be viewed as a reconciliation of the two extreme paradigms. The mechanisms that inhibit immune flares or chronic inflammation in autoimmune diseases and transplantation may be at play in the induction of maternal immune tolerance during pregnancy. Similarly, the pro-tumorigenic mechanisms that allow cancer development to take place may also be responsible for allowing the growth and development of a semi-allogeneic fetus in the mother’s womb. In this review, we investigate the possible mechanisms by which Bregs confer protection to the semi-allogeneic fetus during pregnancy by assessing their role in autoimmune diseases, graft tolerance, and cancer. We also examine how the maternal endocrine system sets the stage for pregnancy, particularly how it endeavors to create an environment conducive for the expansion of Bregs.

## Regulatory B Cells

B cells are best known to organize immune responses by production of antibodies. Representing the humoral arm of adaptive immunity, they are the primary facilitators of antigen-specific immune responses *via* antibody production and differentiation into memory cells that provide long-lasting immunity. However, reports over the past 40 years indicate that not all B cells function for that purpose. The earliest studies (1974) found that B cells could suppress delayed-type hypersensitivity reactions in guinea pigs, implying an inhibitory effect of B cells on T cell function (9, 10). Further evidence of this B cell regulatory phenotype eventuated more than two decades later, with the observation in a murine autoimmune model that inflammation was exacerbated in the absence of B cells (11). While this suggested that B cells may play a down-modulating role in the inflammatory response, it was only in 2000 that Mizoguchi et al. formally described and reported a subset of B cells that inhibited, rather than promoted, the inflammatory response in a mouse model of inflammatory bowel disease (12). This peculiarly suppressive B cell subset was classified as ‘regulatory B cells’ or ‘Bregs.’ Since then, defective Breg function or deficiency in Breg levels have been implicated in conditions involving uncontrolled pro-inflammatory immune responses; most extensively in autoimmune diseases and renal transplantation cases (13–16).

### Breg Phenotypic Identification

Defining a specific Breg phenotype has proven to be a difficult as multiple B cell “subsets” have been reported to function as negative regulators of the immune response. While there is no unifying characteristics with respect to cell surface activation and lineage markers as of yet, initial reports indicated that the regulative properties of these unique B cells were attributed exclusively to the production of the anti-inflammatory cytokine interleukin-10 (IL-10) (13, 17, 18). However, more recent studies have revealed B cell subsets with IL-10-independent regulatory functions, indicating that some Bregs employ a multi-mechanistic, and possibly cooperative, approach for regulating immune responses. Given the lack of a unified approach and as IL-10 production is the most reported mechanism of suppressive action; IL-10 production remains the defining feature of Bregs.

Different B-cell subsets that have been attributed with regulatory function in mice include the transitional 2 marginal-zone precursor (T2-MZP) cells, CD5^+^CD1d^hi^IL-10^+^ B (B10) cells, follicular (FO) B cells, marginal-zone (MZ) B cells, CD5^+^B-1a cells, CD5^+^CD178^+^ killer B cells, GIFT-15 B cells, plasma cells, plasmablasts, TIM-1^+^ B cells, and PD-L1^hi^ B cells (19, 20). In humans, immature B cells, IL-10^+^ B cells (B10), GrB^+^ B cells, Br1 cells, and plasmablasts are reported to play immunosuppressive roles (19). Despite the diversity in phenotype, most B cell subsets that carry out negative regulation produce anti-inflammatory cytokines, with the majority of the cell surface marker-defined subsets enriched with IL-10-producing cells. In mice, the suppressive IL-10-producing Bregs, also known as B10 cells are characterized by the CD1d^hi^CD5^+^ phenotype (21). Among the splenic B10 cells, both marginal-zone B (MZ B) cells and T2-MZP B cells have been shown to have a protective effect in mouse models of lupus and autoimmune arthritis due to their IL-10 competency (22, 23). The peritoneal cavity contains B-1a cells that are also a major source of IL-10 (24). In humans, CD19^+^CD24^hi^CD38^hi^ B cells isolated from human peripheral blood are classified as Bregs due to their ability to suppress inflammation by a combination of IL-10 production and CD80 and CD86 costimulation (25), while the IL-10-competent CD24^hi^CD27^+^ B cells are proposed as the Breg subset analogous to the mouse regulatory B10 cells (26). The heterogeneity of these subsets suggests that Bregs are not derived from one specific lineage; rather they may acquire their regulatory ability through exposure to environmental stimuli.

Since surface markers identifying these subsets are varied, there are currently ongoing attempts to identify a unique global indicator for Bregs, analogous to the transcription factor FOXP3, a defining feature of regulatory T cells (Tregs). Evidence suggests that the T cell immunoglobulin and mucin domain 1 (TIM-1) may be an inclusive marker for Bregs as it identifies about 70% of all IL-10^+^ B cells in all subsets mentioned (27). However, CD9 has also been reported as a marker for murine IL-10-competent Bregs by virtue of transcriptomic analysis which strongly associated its expression with Breg biogenesis and function (28). In principle, identification of a unique marker signifying a direct regulatory function in B cells would reduce the reliance on functional assays and increase our understanding of the role that Bregs may play in different diseases and allow exploration of therapeutic options. To date, elucidating the Breg phenotype and underlying ontology continues to be an active research endeavor.

### Bregs’ Suppression Mechanisms

Since formal recognition, Bregs have been described mainly in the context of autoimmunity, transplantation tolerance, and cancer. The best studied mechanism for negative regulation of Bregs is their capacity to produce anti-inflammatory cytokines, especially IL-10. The principal function of IL-10 is to repress excessive inflammation and maintain homeostasis through numerous mechanisms, which includes hampering antigen presentation and expression of costimulatory molecules CD80, CD86, and MHC class II by macrophages and DCs, as well as limiting the activation of and cytokine release by T cells in immune responses and promoting the differentiation of Tregs (29).

While IL-10 production remains the hallmark of Breg’s negative regulatory function, other independent suppressive mechanisms have come to light in recent years and include transforming growth factor beta (TGF-β), IL-35, and IL-17. Production of TGF-β by B cells leads to promotion of Treg development in diabetes, transplant tolerance, allergic diseases, and colitis (30). Moreover, about 60% of IL-10-producing B cells in mice also express IL-35, a cytokine identified as a key player in the recovery phase of experimental autoimmune encephalomyelitis (EAE), and necessary for improved resistance to *Salmonella* infection (31). Secretion of IL-17 by activated B cells has been reported to occur upon contact with parasite-derived trans-sialidase, a feature shown to be critical in the control and resolution of infection (32). This result emphasizes the importance of Breg function in a disease that has a high potential to develop into chronic inflammation.

Various surface molecules on B cells have also been identified as the main instigators of negative regulation upon cognate interaction with pro-inflammatory cells. Among the most studied are programmed death receptor ligand 1 (PD-L1), granzyme B, and Fas ligand (FasL). Upregulated PD-L1 expression on B cells is critical in down-modulating T cell activity. In EAE, adoptive transfer studies demonstrate that PD-L1^+^ B cells confer protection and reduce disease severity *via* restriction of helper T (Th) cells inflammatory role and induction of Treg activity (33–35). Furthermore, PD-L1^+^ B cells have been shown to regulate cytotoxic T cells in both *Salmonella* infection and prostate cancer (36, 37), with high expression shown to directly suppress the FO Th response and differentiation *via* attenuation of downstream signaling pathways concomitant to programmed death receptor 1 (PD-1) ligation (38). Similarly, B cells expressing FasL were found to be critical in preventing graft rejection in mice and modulating the induction of autoimmune disease through a myriad of mechanisms including promotion of apoptosis of autoreactive T cells and the generation of Tregs (39, 40). Lastly, IL-21-induced granzyme B expression in B cells was shown to impart a regulatory function against effector T cells in the tumor microenvironment (41).

### Breg Detection and Expansion

Due to the inconsistencies in specific surface markers used to classify Breg subsets, the most common approach for Breg identification remains a combination of surface marker staining and intracellular IL-10 staining. In mice, isolated Bregs do not readily produce IL-10 *ex vivo*, therefore to identify this specific subset of B cells, cells are subjected to acute B cell stimulation—5 h of culture with a cocktail of lipopolysaccharide (LPS), phorbol 12-myristate 13-acetate, and ionomycin to enhance the translation of cytokine genes, and thus detectable IL-10 production (42). In the spleen and other lymphoid tissues, Breg progenitor cells have also been identified (43). These are defined as B cells that do not express IL-10 following the short-term stimulation but may be induced to Breg maturation and IL-10 competency by culture with agonistic CD40 monoclonal antibody for 48 h. This technique has proven beneficial for expanding this rare subset for subsequent *in vivo* applications such as adoptive transfer experiments. Together, active and progenitor Bregs account for 3–8% of basal B cell levels in the murine spleen, of which 1–3% are IL-10-competent Bregs. Lower percentages are found in the blood, lymph nodes, intestinal tissues, Peyer’s patches, and the central nervous system (21, 44, 45). In humans, IL-10-producing B cells isolated from the peripheral blood are found to express increased levels of CD19, IgD, CD27, CD48, and/or CD148 at varying proportions and phenotypic combinations. IL-10-competent Bregs have also been identified in peripheral tissues such as spleen, tonsils, and umbilical cord blood at less than 1% of the total B cell population (26).

## Bregs in Autoimmunity

In the last decade, numerous studies have been conducted to determine the operating mechanisms that impart Breg-mediated suppressive function. The majority of what is currently known stems from the multitude of studies focusing on autoimmune diseases where it has been demonstrated that Bregs interact and regulate the function of T cells, DCs, monocytes, macrophages, and natural killer cells.

The regulatory role of B cells in EAE, a mouse model for multiple sclerosis (MS), was first apparent when exacerbation of disease was observed in B cell-deficient B10.PLμMT mice (11). The current model posits that Bregs regulate EAE severity through IL-10 production and expression of costimulatory molecules CD40, CD80, and CD86, thus limiting the type 1 cytokine response *via* cross talk between B cells and T cells, and recruitment of FOXP3^+^ Tregs into the central nervous system (6, 14). B cells expressing toll-like receptor (TLR)-2 and TLR-4 also suppressed inflammatory T cell responses (Th1 and Th17) and stimulated the recovery phase of EAE (46). Other mechanisms such as the presence of glucocorticoid-induced TNF ligand and elevated levels of B and T lymphocyte attenuator on B cells also play a role in inducing and maintaining the Treg pool in the central nervous system during EAE, a process which ultimately facilitates the protection against or amelioration of the disease (47, 48). Interestingly, recent evidence indicates that Bregs can also directly access the central nervous system (CNS). Bone marrow cells transiently stimulated with the TLR-9 agonist CpG generate proB (CpG-proB) cells, which upon transfer to recipient mice at the onset of EAE symptoms differentiate into mature B cells with regulatory function. Some of these mature B cells home to the inflamed CNS for local production of IL-10 thus instigating a switch of the host cytokine profile from inflammatory to immunoregulatory, while others enter reactive lymph nodes and restrain encephalitogenic T cells *via* CCL19 expression. Together these functions cooperatively enhanced amelioration of active EAE (49). Evidence of the regulatory function of B cells in human disease is also accumulating, with B cells from MS patients observed to have diminished capacity for IL-10 production on activation as opposed to healthy controls. This suggests that dysregulation of this pathway contributes to MS pathogenesis (50–52).

Type 1 diabetes (T1D) is characterized by the autoimmune-mediated destruction of pancreatic β cells. Circulating CD19^+^CD27^−^CD24^hi^CD38^hi^ Bregs have been found to decrease with age and as such are hypothesized to be a major contributing factor to the prevalence of T1D in older age groups (53). Patients with T1D also exhibit the lowest frequency of B10 cells compared with patients with type 2 diabetes, latent autoimmune diabetes in adults, and healthy controls (54). These results highlight the importance of B cells in the immunopathogenesis of autoimmune diabetes and suggest that antigen-activated B10 cells may have a role to play in inhibiting autoreactive T cell responses to islet-specific antigen in healthy individuals, as the absence of this population leads to hyperglycemia in T1D patients. In non-obese diabetic (NOD) mice, adoptive transfer of CD1d^hi^CD5^+^IL-10^+^ Bregs prevented T1D development. Further, the addition of tolerogenic DCs to the transferred therapeutic mix reversed the onset of T1D by augmenting the frequency of IL-10^+^ Bregs (55). In the same manner, adoptive transfer of CpG-proB cells, either directly or indirectly after maturation into mature Bregs, protected against the pathogenesis of T1D in NOD mice *via* suppression of IL-21 and induction of apoptosis in effector T cell populations, including specific diabetogenic T cells (56). The DC- and CpG-mediated suppression of T1D through Breg activity reiterates the notion that B cells have an intrinsic suppressive potential, which is only activated when the environmental cues are appropriate.

In systemic lupus erythematosus (SLE), Bregs are emerging as vital players during disease initiation. Adoptive transfer of splenic CD5^+^CD1d^hi^ Bregs from wild-type mice extended the survival period of CD19^−/−^ mice in this spontaneous lupus model (57). In humans, SLE patients exhibited similar frequencies of circulating CD24^hi^CD38^hi^ B cells but had significantly lower IL-10^+^ percentages compared to normal healthy controls (58). Further, there is evidence of functional impairment in Bregs from SLE patients. Isolated Bregs were unresponsive to CD40 ligation and exhibited lower IL-10 production compared to Bregs obtained from normal patients, which resulted in inadequate suppression of T cell activation and proliferation (59). Compromised cross talk between IL-10-producing CD24^+^CD38^hi^ Bregs and interferon alpha-producing plasmacytoid dendritic cells has also been documented as a key contributor to the pathogenesis of SLE (60).

Studies of rheumatoid arthritis (RA) suggest that Bregs are responsible for the primary induction of Tregs. Specifically, IL-10 from B cells aids in the establishment of the Treg to inflammatory Th1 and Th17 cell ratio as seen in chimeric (IL-10^−/−^ B cell) mice which exhibit a significant decrease in FOXP3 expressing CD4^+^ T cells; the low number of Tregs occurred in parallel with an increase in Th1 and Th17 cell populations in the draining lymph nodes of the inflamed joints (61). Furthermore, the adoptive transfer of Bregs into these chimeric mice restored the Treg population to normal levels and decreased inflammation by inhibiting Th1 and Th17 differentiation, thus decreasing IFNγ and IL-17 cytokine levels, and reinstating a balanced Th1/Th2 response (62). This notion of Bregs influencing T cell plasticity was similarly demonstrated by injection of exogenous B10 cells which effectively suppressed the development of arthritis by suppressing Th17 cell generation (63). Clinical studies report decreased frequency of CD24^hi^CD38^hi^ Bregs in RA patients compared to unaffected individuals; it was shown that normal levels of Bregs effectively inhibited CD4^+^CD25^−^ T cell differentiation into Th1 and Th17 cells and promoted the differentiation of T cells into Tregs *via* IL-10 production (64). Moreover, it has been demonstrated that many RA patients achieve remission during pregnancy, presumably due to a shift in T cell function from a Th1 to a Th2 phenotype, but relapse postpartum, a phenomenon possibly correlated to the increase of Breg levels during pregnancy and consequent decrease upon parturition (65–67).

The regulatory function of human B cells in autoimmune diseases is realized in case studies where complete B cell depletion therapy with rituximab (anti-CD20 monoclonal antibody) has resulted in aggravation of the disease or onset of new immune-mediated pathologies. For instance, a patient with intractable ulcerative colitis experienced severe clinical aggravation of disease upon treatment with rituximab, a result ascribed to the depletion of both the intestinal and systemic suppressive B cell population (68). Similarly, rituximab was an effective treatment in a patient with Graves’ disease; however, development of ulcerative colitis and arthritis was observed shortly thereafter. Again, the depletion of colonic B cells was implicated, with symptoms alleviated upon repopulation (69).

Therefore, in autoimmune disease, the suppressive role of Bregs in the framework of T cell function occurs at two levels: (1) control of T cell plasticity by invoking the production of Tregs, inhibiting activation of naïve T cells, and suppressing Th differentiation; and (2) manipulation of the production of proinflammatory and anti-inflammatory cytokines and their balance thereof.

## Bregs in Transplantation Tolerance

Graft tolerance has been best studied in liver and kidney transplantations. Postoperative care involves continuous treatment with immunosuppressive drugs to prevent the immune response to the alloantigen, which may lead to organ rejection. Some recipients require immunosuppression medication for their entire lifetime; however there are reports of operationally tolerant recipients maintaining graft function despite immunosuppressive drug withdrawal (70). The containment of the inflammatory response that typically ensues without immunosuppressive drugs points to the valuable function of regulatory immune cells—the Tregs which have an established role in graft tolerance, and the Bregs, whose role in transplantation has recently emerged.

Initial reports implicating a role for B cells as facilitators of graft tolerance resulted from studies investigating the effect of CD40 blockade on resting B cells. Permanent survival of mouse pancreatic islet allografts was demonstrated upon pretreatment with allogeneic non-T cell small lymphocytes (elutriated from spleen cell suspensions) plus blocking antibody to CD40L (71). Moreover, pretreatment of resting B cells with anti-CD40 monoclonal antibody prolonged fully allogeneic mouse cardiac allografts and augmented the hyporesponsiveness to MHC molecules *in vivo* (72), suggesting a dominant role of the CD40/CD40L pathway in tolerance induction. The mechanism by which anti-CD40 antibodies can elicit a tolerogenic response was elucidated a decade later when it was demonstrated that CD40 ligation resulted in the differentiation of IL-10^+^ transitional-2 (T2) Bregs, the rescue of B cells, and transitional B cell subsets from apoptosis, as well as prevention of differentiation into mature FO B cells. Specifically, anti-CD40 Ab administration induced the differentiation of T2 and MZ B cells and expanded the population of IL-10^+^ Bregs *in vitro* and *in vivo*, resulting in significantly improved renal disease and controlled progression of lupus in mice (73). This was also reflected in tolerant kidney and cord blood transplant patients where increased CD40 ligation potentiated IL-10 production by naïve and transitional B cells (74–76).

In a rat model of kidney allografts, intravenous injection of bulk donor B cells to the recipient at the time of transplantation was by itself proven to be effective in inducing long-term acceptance (more than 300 days) of the graft. This was a significantly longer time period than when donor T cells were administered 17 days after transplantation (77). Studies of islet allografts in mice identified TIM-1 as an inclusive marker for IL-10 and IL-4 expressing Bregs in all major B cell subpopulations including the transitional, MZ, FO, and CD5^+^CD1d^hi^ cells. More importantly, ligation by anti-TIM-1 induced the regulatory function of these B cells constitutively expressing TIM-1 *via* IL-10 production. Adoptive transfer of TIM-1^+^ B cells from untreated BALB/c allograft recipients into chemically diabetic B cell-deficient allograft recipients prolonged graft survival by activation of transferred IL-10-producing TIM-1^+^ B cells and augmented the frequency of FOXP3^+^ Tregs (27). Moreover, the mucin domain of TIM-1 has been identified as being primarily responsible for Breg induction and maintenance in prolonged allograft survival (78). Aside from TIM-1^+^ Bregs, tolerance in a mouse model of MHC class I mismatched skin transplantation was associated with a transient expansion in T2 B cells in mice tolerized by donor splenocyte transfusion and ligation with CD40L. These tolerized B cells were characterized by expression of downregulated levels of CD86 and upregulated levels of TIM-1, and prolonged skin allograft survival *in vivo*, as well as suppressed T cell activation *in vitro*. Moreover, it was seen in this study that tolerized B cells and graft-specific Tregs worked synergistically in prolonging graft survival, albeit in an IL-10-independent fashion (79). Similarly, the adoptive transfer of TGF-β-producing Bregs induced the expression of FOXP3 in CD4^+^CD25^−^ T cells, thus activating and expanding the Treg population which contributed to protracted graft survival (80). These reports reinforce the multi-mechanistic mode of suppression by Bregs in that through IL-10, TGF-β, CD86 costimulation, and other surface molecules, antigen-specific tolerized B cells no longer stimulate allospecific T cell activation, but instead support the induction of Treg function to enact a tolerogenic response.

In humans, research has also indicated a critical role for B cells in regulating alloimmunity. For operationally tolerant renal transplant recipients, renal allograft tolerance was strongly associated with a B cell signature consisting of an increased expression of multiple B cell differentiation and activation genes over that observed in non-tolerant patients receiving immunosuppression and non-transplanted controls. The three genes identified as reliable predictors of operational tolerance—*IGKV4-1, IGLL1*, and *IGKV1D-13*—are genes that are upregulated during transition from pre-B to mature B cells and during class switching post-antigen stimulation, suggesting that transitioning B cells may be highly involved in tolerance induction and/or maintenance (15). Moreover, operationally tolerant patients displayed higher transitional (CD24^hi^CD38^hi^) and naïve B cell frequencies, lower numbers of memory B cells, and an enriched IL-10^+^ B cell population—all phenotypes that were strongly associated with reduced allograft rejection rates (81–84). Together these recent findings suggest assigning a prospective biomarker status on transitional B cells. Additionally, operationally tolerant patients retain the capacity to activate the CD40 pathway essential to Breg activation (16), and although IL-10-producing Bregs are higher in these patients, other mechanisms such as granzyme B^+^ and TGF-β production may also account for the inhibitory effect on T cells essential to maintaining transplantation tolerance (80, 85).

## Bregs in Cancer

Oncogenesis is primarily a battle between the immune system and the cancer stem cells. Tumor development and progression is dependent on the lenience of the microenvironment, to which leukocytes play a critical role. Both anti-tumorigenic and pro-tumorigenic immune mechanisms transpire in the early stages of cancer development, with the net effect dictating whether the tumor develops or not (86). In particular, the induction of immune tolerance within the tumor environment has been shown to curtail immune-enhancing anti-tumorigenic efforts and encourage tumor progression and subsequent metastases. Immunosuppressive cells such as Bregs and Tregs have been implicated in facilitating immune tolerance, and ultimately, cancer escape.

For instance, tumor growth is contingent on the immune profile within the local microenvironment. Strong infiltration of antitumor CD8^+^ T cells and natural killer cells and decreased levels of regulatory B and T cells and myeloid-derived suppressor cells (MDSCs) all result in tumor destruction, whereas reversed proportions support tumor growth. In the tumor-draining lymph nodes, increased accumulation of B and T cells resulted in lymphangiogenesis and increased lymph flow (87), which contributed to and accelerated the rate of metastasis (88). Moreover, B cell accumulation in lymph nodes of preneoplastic Eμ-*c-myc* mice was positively correlated with expansion of the lymphatic sinuses and enhanced tumor growth, as opposed to preneoplastic B cell-deficient μMT mice which did not undergo lymphangiogenesis and exhibited minimal tumor growth (89). However, tumor growth was accelerated in the B cell-deficient μMT mice upon adoptive transfer of naïve T2-MZP cells, highlighting the role of transitional and suppressive B cells in cancer escape. Furthermore, B cells bearing downregulated MHC class II and CD86 expression and upregulated Ly6A/E, PD-L1, and CD39 selectively accumulated in the draining lymph nodes of mice with HPV-related cancer. The presence of PD-L1 and CD39 on the B cell surface confers regulative properties *via* direct inhibition of T cell function upon cognate interaction. The depletion of B cells in this model resulted in a robust Th1 cytokine response and decline in recruitment of Tregs, which was accompanied by a strong infiltration into the tumor of CD8^+^ T cells and ultimately tumor rejection (90). Interestingly, although primary colorectal tumors contain a significant memory and plasma B cell infiltrate suggestive of an active antitumor response, CD24^hi^CD38^hi^ Bregs were significantly increased in metastatic tissue indicating a shift toward active immunosuppression in the local microenvironment (91). Thus the current literature suggests that B cell infiltration and accumulation in the tumor site and draining lymph nodes establishes an immunosuppressive microenvironment that leads to inhibition of antitumor immune responses and expansion of the critical lymphatic network, thereby facilitating tumor growth and metastasis.

Investigations into the specific role of Bregs in cancer have recently increased following the discovery of a unique B cell subset termed tumor-evoked Bregs (tBregs) in Balb/c mice harboring 4T1 carcinoma cells (8). In this model it was shown that cancer-eradicating B cells were ‘hijacked’ by resident cancer cells within the tumor microenvironment and converted into immunosuppressive Bregs. These tBregs do not conform to any of the established Breg phenotypes; instead they resemble activated mature B2 cells (CD19^+^CD25^hi^CD69^hi^ and B7-H1^hi^CD81^hi^CD86^hi^CD62L^lo^IgM^int^). Incubation of non-regulatory CD4^+^ T cells with tBregs bearing high expression levels of CD40, CD80, CD86, MHC class I and II molecules, and TGF-β production capacity led to the significant generation of CD4^+^CD25^+^FOXP3^+^ Tregs which in turn inhibited CD8^+^ T cell proliferation—thereby facilitating breast cancer escape and metastasis (8). More recently, it has been demonstrated that B cells infiltrating the mammary tumor bed are ‘educated’ through cognate interactions with resident tumor cells. These tumor-infiltrating B cells acquired PD-L1 expression and TGF-β competency which contributed to an immunosuppressive phenotype characterized by an enhanced inhibitory capacity against CD4^+^CD25^−^ T cells, CD8^+^ T cells, and CD49b^+^NK cells, as well as promotion of Treg expansion (92). Therefore, infiltrating B cells subverted into Bregs by the tumor microenvironment facilitates the necessary critical changes in the local immune profile to support a pro-tumorigenic outcome.

In humans, studies reveal that a higher frequency of Bregs is indicative of enhanced tumor aggressiveness and poorer prognosis. For instance, hepatocellular carcinoma patients harbor significantly more peripheral Bregs compared to healthy controls (93); malignancy in non-small cell lung cancer is associated with an increased frequency of IL-10-producing Bregs, CD4^+^CD25^+/high^CD127^low/−^ Tregs, and MDSCs (94); patients with tongue squamous cell carcinoma, gastric cancer, and colorectal cancer also exhibit a higher frequency of Bregs within the tumor itself compared to unaffected neighboring tissues, with numbers positively correlated with an increased frequency of Tregs (91, 95, 96). Moreover, *in vitro* studies of lung cancer cells demonstrated their direct capacity to upregulate Treg and Breg phenotypes in lymphocyte cocultures (94). As for the mechanism, IL-10 and TGF-β competency appear to be the primary facilitators of suppression. For example, peripheral blood CD19^+^CD24^hi^CD38^hi^ Bregs isolated from gastric cancer patients converted CD4^+^CD25^−^ effector T cells into CD4^+^FOXP3^+^ Tregs *via* expression of TGF-β (95). Moreover, IL-10 facilitates cross talk between CD19^+^IL-10^+^ Bregs and CD4^+^CD25^−^ T cells in tongue squamous cell carcinoma patients, resulting in the conversion of these resting T cells into Tregs (96). Likewise, IL-10 has been shown to induce the expansion of Tregs in peripheral lymphoid organs in gastric cancer patients (95).

## Bregs and Female Sex Hormones

The two major sex hormones involved in pregnancy are estrogen and progesterone, with each hormone playing a central role at different time points during gravidity. Progesterone promotes endometrial decidualization for the implantation of the embryo and maintains uterine relaxation throughout pregnancy (97). Estrogen on the other hand, plays a central role in angiogenesis which is needed for placentation and sustenance of the fetus (98). High estrogen levels and the withdrawal of progesterone promote the onset of labor, and ultimately, human parturition. Female sex hormones were found to have a significant influence on inflammatory responses when women with autoimmune conditions were reported to experience a change in their disease activity upon pregnancy. For instance, women that acquired SLE during pregnancy generally experienced a more severe affliction compared to non-pregnant patients (99, 100). Existing RA is often ameliorated by the surge of immunoinhibitory estrogen during pregnancy, which relapses postpartum upon an increase in the immunostimulatory hormone prolactin (101, 102). There is also evidence that female sex hormones are capable of modifying decidual immune cells, such as DCs and uterine natural killer cells, from being actively pro-inflammatory to being tolerogenic (103). Considering that pregnancy outcomes essentially rely on the effective control of the inflammatory response, essential pregnancy hormones should be investigated in any study of immune cell changes during pregnancy.

Estrogen regulation of immune cells, whether innate or adaptive, has been established in recent years. Its presence can increase splenic neutrophil numbers, alter the phagocytic capacity of macrophages, and enhance the maturation of DCs (2). It has also been shown to regulate the activities of CD4^+^ and CD8^+^ T cells and promote the expansion and activity of Tregs by increasing FOXP3, PD-1, and cytotoxic T-lymphocyte-associated protein 4 expression (34, 104, 105). RA and MS can both be ameliorated by estrogen-induced Treg expansion and activation which leads to immune suppression (104, 106). B cells are likewise affected by the presence of estrogen. During lymphopoiesis, a rise in estrogen levels leads to a reduction of B cell precursors and an expansion of mature B cells. Estrogen activation therefore affects B cell maturation, and ensures that the immune system is equipped to defend the body against pathogens if required (5).

Two receptors are responsible for estrogen-mediated signaling—estrogen receptor α (ERα) and estrogen receptor β and upon activation act as transcription factors for a range of estrogen-sensitive genes, with ERα more predominant on B cells (107). Studies of EAE demonstrate that the protective effect of 17β-estradiol (E2) comes from its interaction with ERα on immune cells, including B cells (106). While many studies have explored the protective effect of estrogen in autoimmune diseases, primarily by focusing on effects on Tregs (34, 108), others have highlighted the role of B cells by showing that E2 conferred protection against the induction of EAE in mice even after ablation of Treg; a result unsuccessful in B cell-deficient mice (6, 109). Specifically, E2 augmented the frequency of splenic IL-10^+^ Bregs *via* the PD-1/PD-L1 mechanism (110, 111) which led to a decline in the frequency of CD11b^+^CD45^hi^-activated macrophages, DCs, and infiltrating CD4^+^ cells in the spinal cord (112). Together, these results suggest that Bregs may have carried out this protective effect *via* their responsiveness to estrogen. This was confirmed by studies which demonstrated that estrogen-receptor positive B cells upregulate Treg function in EAE (34).

The effect of progesterone on innate immune cells, mainly monocytes, DCs, and natural killer cells, has also been elucidated (103), and in a manner similar to estrogen, depends on the presence of progesterone receptors. These receptors also act as nuclear transcription factors upon activation and may either be progesterone receptor A (PR-A) or progesterone receptor B (PR-B) (113). The specific actions of progesterone relevant to myometrial function are mediated by the combined and distinct genomic actions of PR-A and PR-B. Myometrial cells express PR-B for most of the pregnancy, facilitating progesterone-mediated Th2-like maternal immune responses, resulting in reduced production of pro-inflammatory cytokines, and increased secretion of IL-10 (97, 114). At the latter part of pregnancy nearing the onset of parturition, PR-A gene expression becomes more predominant, blocking the transcriptional anti-inflammatory activity of PR-B and consequently increasing pro-inflammatory gene expression and promoting labor (97). Progesterone function in cancer and autoimmune diseases occurs comparably to that in pregnancy, in that progesterone inhibits T cell proliferation and pro-inflammatory cytokines IL-2, IL-17, and IFNγ expression while enhancing IL-4, IL-5, IL-6, and IL-10 cytokine production (98, 115). Moreover, there is a concurrent expansion of B cells associated with the increase in IL-10, and a lack of effect of progesterone on Tregs (116). Collectively, this suggests a possible signaling pathway wherein progesterone elicits IL-10 augmentation by activating Bregs.

The molecular mechanisms by which estrogen and progesterone operate appear to be distinct from one another. Whereas estrogen elicits a strong effect on Tregs, progesterone does not. However, both have been documented to affect the B cell immune profile. The current literature gives insights as to how the female sex hormones shape the immune response in various clinical conditions, and overall indicate that estrogen and progesterone foster a tolerant environment within the pregnancy milieu by expanding all regulatory cells. Changes in the circulating amount and distribution of these cells during pregnancy indicates that adaptations are in place to ensure that the mother’s body is well equipped to conceive and grow a semi-allogeneic fetus.

## Bregs in Pregnancy

Previous studies of Bregs in the context of autoimmune disease, graft transplantation, and cancer all indicate one thing—Bregs dampen the inflammatory response and foster a stable tolerant immune profile within the local microenvironment. Based on the current literature, it is therefore reasonable to speculate that Bregs may be a key player in pregnancy. Their anti-inflammatory role in autoimmunity and graft tolerance suggest relevance to the induction of maternal immunological tolerance, while their pro-tumorigenic role in cancer may be applicable in pregnancy, i.e., maternal–fetal cognate interactions may hijack naïve B cells and educate them to become Bregs thus ensuring the fetus’ unperturbed growth. The pregnancy hormones estrogen and progesterone are likewise critically involved in the establishment, maintenance, and termination of pregnancy.

During pregnancy, as the mother’s womb houses and protects the semi-allogeneic fetus, the response of the maternal immune system is the key determinant to pregnancy success, with failure to induce selective immune tolerance toward the fetus resultant in pregnancy loss. Modification of the maternal immune response is orchestrated by multiple cytokines that influence the nature and abundance of leukocyte subsets in the uterus and placenta and includes the reduction of antigen-presenting function of monocytes, macrophages, and DCs; inhibition of natural killer cells, T cells, and B cells; proliferation of uterine killer cells; maintenance of tolerogenic DCs; and the induction of Tregs (117). These immunological adaptations during early pregnancy pave the way for two main objectives—to protect the fetus from immune rejection and to facilitate the tissue remodeling processes needed for placental development (118).

Primarily, Bregs are considered highly relevant in the pregnancy milieu as they are a major cellular source of the potent anti-inflammatory cytokine IL-10. This cytokine is vital for optimal pregnancy outcomes, with IL-10 deficiency associated with fetal resorption, growth restriction, and even death (118–120). IL-10 is found in abundant amounts in the uterus and placenta during pregnancy and has been identified as a critical player for counteracting the pro-inflammatory cytokine response. Even at the initial conception stage, inflammation resulting from the recognition of paternal antigens is thwarted by the presence of IL-10 (121). Furthermore, administration of a sub-clinical dose of LPS to pregnant IL-10^−/−^ mice resulted in a 10-fold reduction in viability of semi-allogeneic fetuses compared to unaffected controls. Analysis of maternal serum and uterine tissue in these LPS challenged IL-10^−/−^ mice demonstrated high levels of the pro-inflammatory cytokines TNFα, IL-6, IL-1A, and IL-12p40, a result which could be abrogated by administration of exogenous IL-10 (118, 122, 123). Therefore, IL-10 confers pregnancy protection by multiple mechanisms including inhibition of pro-inflammatory cells such as macrophages and monocytes, and suppression of pro-inflammatory cytokine and chemokine production such as TNFα, IL-6, IL-1a, IL-8, and IL-12 (118, 124, 125). These findings demonstrate the capacity of IL-10 to regulate the inflammatory response in the uterus and placenta during gestation.

Evidence of a direct role for Bregs was indicated in comparative mouse studies of normal and abortion-prone pregnancies which showed a diminished frequency of B10 cells in the maternal spleen of abortion-prone mice, whereas normal pregnancies harbored an increased number of B10 cells. Furthermore, adoptive transfer of B10 cells rescued pregnancies in abortion-prone mice, by inhibition of DC maturation and expansion of the Treg population (126). Another Breg phenotype reported as critical to pregnancy success are the MZ B cells that are found expanded in the spleen of normal pregnant mice, but lacking in mice undergoing pregnancy complications. Additionally, successful pregnancy was correlated with the capacity of MZ B cells to produce enhanced levels of immunoglobulin (IgM and IgA) which likely shifts the immune response from a Th1-like to a Th2-like profile (127). Although also IL-10 competent (22), there is no evidence yet linking MZ B cell cytokine production to their role in pregnancy well-being. In humans, IL-10-producing CD19^+^CD24^hi^CD27^+^ B cells in the peripheral blood have been shown to be significantly higher in women undergoing normal pregnancies as opposed to non-pregnant women, or women who had suffered spontaneous abortions (128). Bregs isolated from peripheral blood of pregnant women taken during the first trimester successfully inhibited TNFα secretion by activated T effector cells *ex vivo* (128). Interestingly, CD24^hi^CD38^hi^ Bregs were found to be significantly lower during the third trimester of pregnancy and on delivery day compared to non-pregnant and postpartum controls, possibly as a result of a drop in female sex hormones that influence B cell activation (129). Although limited in number, these studies on Breg function in pregnancy, and the underpinning Breg-mediated immunosuppression, parallels that observed in autoimmune disease, graft tolerance, and cancer. In all conditions, Bregs impose a suppressive environment *via* induction and maintenance of Tregs, modification of the Th response, and inhibition of effector cell responses including cytolytic T cells, NK cells, and DCs.

As Breg function in pregnancy appears to be interlinked with that of Treg and DCs, defining the working mechanisms is requisite. Both Tregs and DCs are critical players in determining pregnancy outcomes. In normal pregnancy, the majority of decidual DCs remain immature which contributes to the maintenance of a fetal-tolerant local environment. These immature DCs are thus referred to as tolerogenic DCs (130). Tregs are likewise crucial contributors in achieving and maintaining maternal–fetal immune tolerance. In mice, a natural periodic accumulation of Tregs during the estrus stage is seen, purportedly in preparation for the implantation of a semi-allogeneic fetus (131). At the onset of embryo implantation, Tregs are recruited to the para-aortic lymph nodes draining the uterus (132). Expansion of the Treg population is critical in improving the rates of successful pregnancy as deficiency or harboring insufficient numbers of Tregs in the vicinity of the uterus at any time during pregnancy leads to abortion or miscarriage (4, 133). In the context of pregnancy, tolerogenic DCs and Tregs foster a fetal-tolerant environment *via* production of anti-inflammatory IL-10 and TGF-β (134).

**Figure 1:**
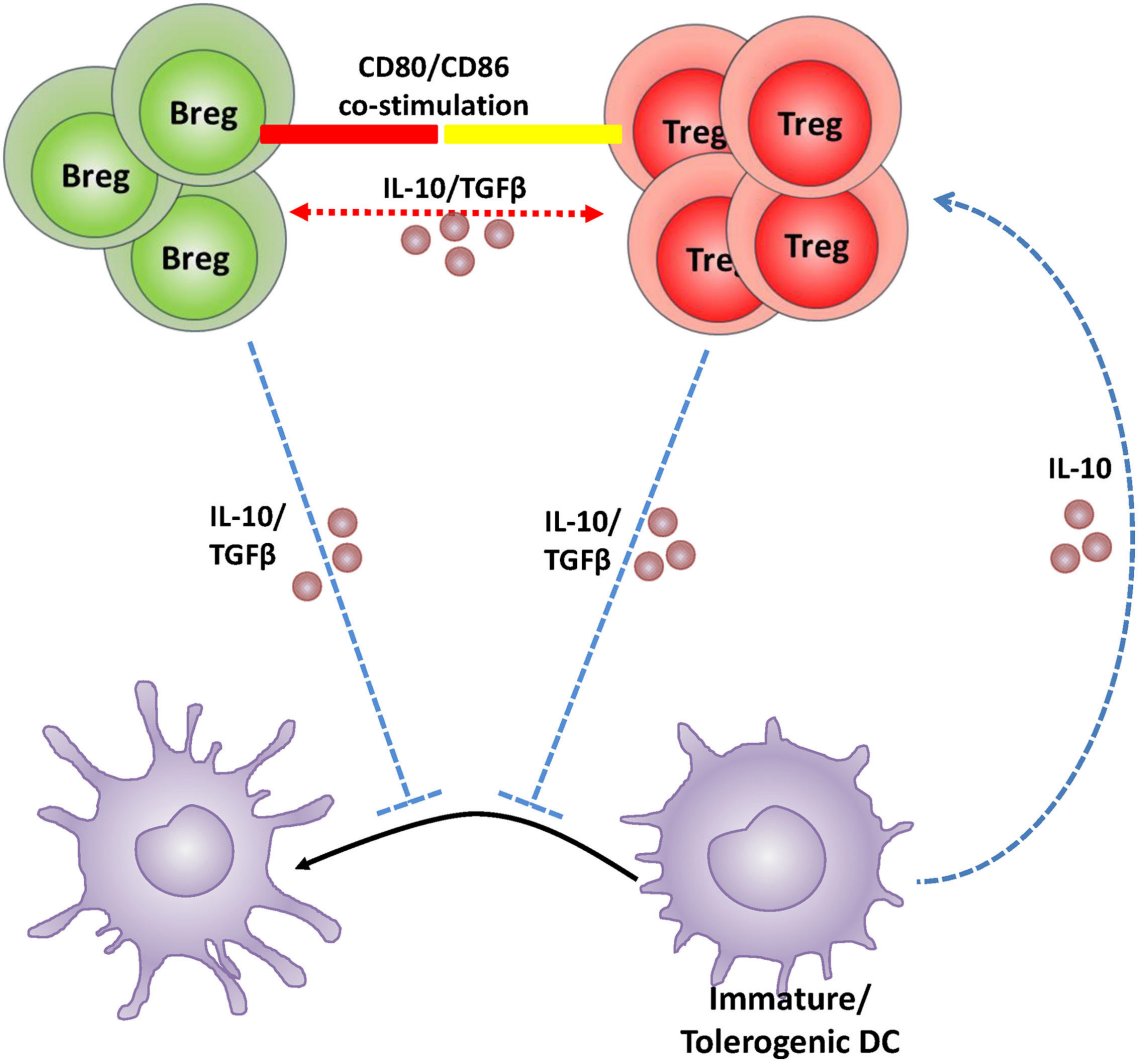
**Schematic illustration of the hypothetical feedback loop between regulatory B cell (Breg), regulatory T cell (Treg), and dendritic cells (DCs) during pregnancy**. Bregs create a tolerant pregnancy milieu either by direct induction and expansion of the Treg population *via* CD80/CD86 costimulation and/or IL-10 and TGFβ production (depicted in red); or by inhibition of DC maturation causing retention of a tolerogenic phenotype and in turn expansion of the Treg population *via* IL-10 (depicted in blue). Tregs and tolerogenic DCs produce IL-10 and may maintain the feedback loop responsible for negative regulation within the pregnancy milieu. The exact mechanism by which Bregs recruit Tregs and inhibit DC maturation in the pregnancy context is, however, yet to be established.

During pregnancy, if an anti-inflammatory signal such as IL-10 or TGF-β is lacking, the DC phenotype remains such that it prevents the activation of T cells that protect the semi-allogeneic fetus (135). It is thus pertinent that DCs retain their immature state to acquire the tolerogenic phenotype. IL-10 and or TGF-β must then be initially present in the pregnancy milieu to either set off the induction of tolerogenic DCs followed by the induction of Tregs, or directly drive the induction of Tregs. However, given that Bregs are (1) established sources of both IL-10 and TGF-β, (2) significantly increased in numbers during normal pregnancy, and (3) are responsive to endocrine modulation, it is plausible that Bregs also play a critical role in pregnancy, especially in the initial induction of maternal immunological tolerance. In support, the findings of Jensen et al. (126) suggest that IL-10 production from B10 cells likely influences the non-maturation of IL-10-receptor rich DCs which then expand the Treg population in pregnant mice. Based on the current literature indicating similar immunosuppressive roles and direct interface among these three types of immune cells, a Breg–Treg–tolerogenic DC feedback loop is proposed for the maintenance of a tolerant pregnancy milieu (illustrated in Figure 1).

## Conclusion

The function of Bregs in autoimmune diseases, graft tolerance, and cancer sheds light as to their emerging role in pregnancy. Studies of autoimmune disease and graft tolerance indicate that the immune response attempts to regain homeostasis by Breg-mediated negative regulation. In cancer, tumor-educated Bregs represent an adaptation that encourages a mutated self to survive and proliferate. Integrating these fields, it is reasonable to suggest that pregnancy represents a predicament common to both—the need for constant immune tolerance for the duration of the pregnancy, and the permission to allow a semi-allogeneic fetus to grow within. However to date, the direct role that Bregs play in maternal tolerance in pregnancy has not been fully investigated, and is the subject of ongoing investigations in our laboratory. Supporting studies suggest that the pregnancy sex hormones, estrogen and progesterone, may also be critical prerequisites for Breg activation and function, with a Breg–Treg–tolerogenic DC feedback loop potentially underpinning the induction and maintenance of a tolerant environment necessary for a successful pregnancy. Bregs therefore, are potential guardians of pregnancy well-being, and require further exploration as to their role in this context.

## Author Contributions

RG-G researched and wrote the manuscript. KD conceptualized the investigation and edited the manuscript.

## Conflict of Interest Statement

The authors declare that review of the literature was conducted in the absence of any commercial or financial relationships that could be construed as a potential conflict of interest.
